# Microbiological Characteristics of Beef Sausage Enriched With Roselle ( *Hibiscus sabdariffa* L.) Sepal Extract

**DOI:** 10.1002/fsn3.4582

**Published:** 2024-11-10

**Authors:** Sharif Ghorbani, Sara Jafarian, Mahdi Sharifi Soltani, Leila Roozbeh Nasiraie

**Affiliations:** ^1^ Department of Food Science and Technology Nour Branch, Islamic Azad University Nour Nour Iran; ^2^ Department of Veterinary, Chalous Branch Islamic Azad University Chalous Iran

**Keywords:** beef sausage, encapsulation, microbiological properties, roselle extract

## Abstract

Adding plant extracts to sausage and other meat products is very important to improve their quality, safety, and durability. The aim of this study was to evaluate the microbiological properties of beef sausage enriched with roselle ( *Hibiscus sabdariffa* L.) sepal extract. The total content of phenolic and flavonoid compounds in hydroalcoholic extract of Roselle sepals was measured by Folin‐Ciocalteu test and aluminum chloride colorimetric method, and antioxidant activity was measured by DPPH method. Pectin was used to encapsulate the extract. The characteristics of capsules, including particle size, zeta potential, and capsule efficiency were measured. Minimum Inhibitory Concentration (MIC) and Minimum Bactericidal Concentration (MBC) of extracts were performed by tube dilution method. Microbial tests were performed on sausage treatment samples. Analysis of the obtained data was done using SPSS and Excel software. The study revealed that the total phenolic and flavonoid compounds in the extract were 174.6 mg of gallic acid per gram and 16.8368 mg of quercetin, respectively. The extract displayed the highest antioxidant activity at elevated concentrations. Particle size ranged from 16.833 to 640.534 μm. The zeta potential and capsule efficiency were found to be satisfactory. Compared to the encapsulated extract, the free roselle extract better inhibited *Staphylococcus aureus* (*s. aureus*), *Salmonella typhimurium* (*S. typhimurium*), and *Escherichia coli* (*E.coli*). During storage until day 20, these bacteria did not grow in the free or encapsulated extract groups. However, *S. aureus* appeared in the free extract group from day 25 and the encapsulated extract group from day 20. Roselle extract has antimicrobial properties that can improve the quality and safety of beef sausage during storage.

## Graphical abstract

The use of roselle extract can be considered a natural and effective solution for enhancing the quality and safety of beef sausage.

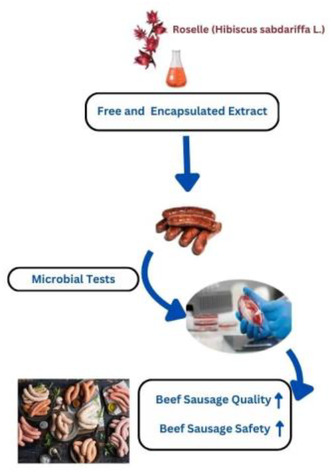

## 1 Introduction

Sausage is one of the widely consumed meat products that has gained popularity in various cultures due to its convenience, variety, and affordability (Azizkhani and Tooryan 2015). With increasing consumer demand for high‐quality and healthy meat products, the addition of natural ingredients to sausages can offer health benefits. Artificial additives, which are commonly used in meat products like sausages for extended shelf life and enhanced color, have been associated with various diseases such as cancer and diabetes (Abdolghafour and Saghir 2014). In order to replace harmful substances and maintain the quality of meat protein, plant extracts and powders can be utilized, particularly in sausage preparation. The use of these plant‐based ingredients not only provides functional compounds but also garners attention due to their health benefits. Roselle, obtained from the sepals of the plant *H. sabdariffa* L. belonging to the Malvaceae family, is prepared. With over 300 species distributed in tropical and subtropical regions worldwide, such as Africa, Central America, South America, Asia, and the Middle East (Nouri et al. 2019; Riaz and Chopra 2018), the red‐colored sepals of the roselle plant are used in the food industry as flavoring and coloring agents, as well as in herbal medicine and is employed as a food supplement, offering numerous health and medicinal benefits (Da‐Costa‐Rocha et al. 2014). Roselle extract, as a rich source of phenolic and flavonoid compounds, has strong antioxidant properties that can increase the oxidative stability and shelf life of meat products. Also, this substance has strong antimicrobial properties that can be effective in controlling the growth of bacteria and increasing the safety of meat products.

Encapsulation is a technology that is particularly suitable for high‐value compounds, and it has garnered significant attention in various industries, including the food, pharmaceutical, and cosmetic sectors, due to its ability to preserve and enhance the biological activity of natural extracts (Vinceković et al. 2017). The encapsulation and controlled release of active substances in food have crucial applications in the food industry, allowing for the maintenance and regulation of temperature, humidity, and other conditions for the materials enclosed within the capsules. This, in turn, leads to increased stability, extended shelf life, and improved food quality (García, Forbe, and Gonzalez 2010; Neethirajan and Jayas 2011).

In meat products, particularly sausages, the use of antioxidant and antimicrobial preservatives is widespread to prevent lipid oxidation, spoilage, and enhance product quality. Incorporating these natural preservatives helps safeguard products from harmful effects. Plants, owing to their diverse array of special compounds, possess antioxidant, antimicrobial, anticancer, and antimutagenic properties (Aminzare et al. 2019; Nikmaram et al. 2018). I. Borrás‐Linares et al. (2015) demonstrated that various extracts of roselle exhibit antioxidant and antimicrobial properties. Research findings indicate that the antioxidant and antimicrobial activity of roselle extracts is influenced by different extraction methods, offering potential as a source for valuable therapeutic products (Jung, Kim, and Joo 2013). A study on the antimicrobial activity and physicochemical properties of a film based on potato starch containing acetic and methanolic extracts of *H. sabdariffa* in sausages revealed the antimicrobial activity of acetic and methanolic extracts of *H. sabdariffa* (Cruz‐Gálvez et al. 2018). In another study, Feridoni and Shurmasti (2020) demonstrated that encapsulation enhances the antioxidant properties of roselle extract, and when encapsulated with CMC, it can delay oxidative spoilage in chicken nuggets. Conversely, Jamhari, Dewi, & Setiyono, (2019) found no interaction between the level of roselle sepal extract and the ratio of wheat flour to mokaf flour in relation to the physicochemical characteristics and antioxidant activity of chicken sausage.

Findings of experimental studies suggest that roselle extract, due to its rich content of phenolic and flavonoid compounds, exhibits strong antioxidant and antimicrobial properties that can enhance the quality and shelf life of meat products (Borrás‐Linares et al. 2015; Jung, Kim, and Joo 2013). Several studies have evaluated the use of free and encapsulated roselle extract in various meat products, such as sausages and chicken nuggets, and have generally found that the incorporation of roselle extract, especially in its encapsulated form, can improve the oxidative stability and microbial safety of the meat products (Bahrami Feridoni and Khademi Shurmasti 2020; Cruz‐Gálvez et al. 2018). However, the existing evidence is not entirely conclusive, as some studies have reported that roselle extract may lack significant antioxidant and antimicrobial effects in certain meat products (Jamhari, Dewi, and Setiyono 2019). Moreover, the majority of the research has focused on the application of roselle extract in poultry‐based meat products, while the effects on beef‐based sausages have been relatively unexplored. This study aims to address these gaps in the current literature by evaluating the impact of both free and encapsulated roselle sepal extract on the microbial characteristics of beef sausages, which could have important implications for improving the quality and safety of these widely consumed meat products.

## 2 Materials and Methods

### 2.1 Preparation of Roselle Plant

Roselle sepal (*H. sabdariffa* L.) were obtained from cultivation in Fanuj area, located in Nikshahr, Sistan, and Baluchistan province of Iran and then sepals were validated by the Technology Development Center of Tehran University of Medical Sciences subsequently. Sepals were dried at 30°C ± 2°C in sunny conditions and kept in aluminum bags at 3°C ± 1°C until consumption (Jafarian et al. 2014). Afterward, the dried sepals were thoroughly ground using a grinder and stored at a temperature of 25°C until further testing. The drying conditions of roselle extract, such as temperature, light, and humidity, can have a positive effect on its final quality. A temperature of 30°C ± 2°C is suitable and prevents the destruction of sensitive compounds. Drying in sunny conditions can preserve some important compounds, such as phenolic compounds. Also, low environmental humidity can lead to a decrease in moisture in the dried extract and maintain its quality. Accurate control of these factors can help maintain the desirable properties of roselle extract.

### 2.2 Isolation of Extracts

*Hibiscus sabdariffa* L. sepal powder was mixed with a 5% ethanol–water solution at a ratio of 1:5 (20 g of sample with 100 mL of solvent). The mixture was then placed in a shaker (Labtron Ls‐100; Iran) and shaken at a speed of 160 rpm for 48 h, away from light. After that, the mixture was subjected to three rounds of centrifugation (10 min each time at 3000 rpm) (z200A; Hermle, Germany). During each centrifugation step, the aqueous phase (supernatant phase) was carefully collected, ensuring no sediment remained at the bottom of the tube. The collected aqueous phases were then filtered through Whatman paper No. 1, and the solvent was evaporated using an evaporator (TAM 2times; Iran) (maximum temperature 50°C) to obtain the roselle sepal extract in the mentioned solvent. The resulting extracts were stored at −18°C until the experiment (Esmaeilzadeh Kenari, Mohsenzadeh, and Amiri 2014).

### 2.3 Total Phenolic Content Determination

The amounts of phenolic compounds in the extract were determined using the Folin–Ciocalteu method (Esmaeilzadeh Kenari Mohsenzadeh, and Amiri 2014), and the results were expressed as mg/g gallic acid equivalent (GAE). For this analysis, 0.5 mL of a 0.1% extract solution (prepared by dissolving 0.1 g of extract in 100 mL of solvent) was mixed with 2.5 mL of Folin–Ciocalteu reagent diluted 10 times and 2 mL of a 7.5% sodium carbonate solution. The samples were then kept at room temperature for 30 min, and at the end of the incubation period, the absorbance was measured spectrophotometrically (T80, PG Instrument; England) at a wavelength of 760 nm (Esmaeilzadeh Kenari, Mohsenzadeh, and Amiri 2014).

### 2.4 Total Flavonoid Content Determination

A colorimetric method was used to determine the flavonoid content. For this analysis, 0.5 mL of the extract (with a concentration of 50 mg/mL) was mixed with 1.5 mL of methanol, 0.1 mL of 10% aluminum chloride, 0.1 mL of 1 M potassium acetate, and 2.8 mL of distilled water. The mixture was left at room temperature for 30 min. Afterward, the absorbance of the reaction mixture was measured at 415 nm using a UV–vis microplate spectrophotometer (MultiskanTM GO, Thermo Scientific, Waltham, MA, USA). A calibration curve was created using quercetin solutions in methanol with concentrations ranging from 10 to 100 μg/mL. The total flavonoid content was expressed as mg of quercetin equivalent per 100 g of roselle sepals (mg QE/100 g dc) (Builders et al. 2013).

### 2.5 Free Radical Scavenging Capacity of DPPH Radical

The capacity to scavenge DPPH free radicals was determined following the method described by Esmaeilzadeh Kenari Mohsenzadeh, and Amiri (2014). In this assay, 0.3 mL of each extract at various concentrations was mixed with 3.7 mL of a DPPH radicals solution (6 × 10^−5^ mol/L). The mixture was vigorously shaken and kept in a dark place for 30 min until the absorbance values reached a steady state. The reduction in DPPH radicals was measured by monitoring the decrease in absorption at 517 nm. The DPPH scavenging effect was calculated as a percentage of DPPH discoloration using the following equation:

$$\%\text{Scavenging effect}=\left(\left[\text{ADPPH}-\mathrm{AS}\right]/\text{ADPPH}\right)\times 100$$

where AS represents the absorbance of the solution when the extracts were added at different concentrations, and ADPPH is the absorbance of the DPPH solution. Methanolic solutions of TBHQ (2‐(1,1‐dimethylethyl)‐1,4‐benzenediol) were used as standards (Esmaeilzadeh Kenari, Mohsenzadeh, and Amiri 2014).

### 2.6 Encapsulation of Roselle Extract

Pectin was selected as the coating material for the preparation of nanoencapsulated roselle extract, following the method described by Bahrami Feridoni and Khademi Shurmasti (2020). As a natural and nontoxic polysaccharide, pectin has the ability to cover and create semipermeable membranes, which can easily dissolve in aqueous solutions and effectively encapsulate the active ingredient. It also has biocompatibility and biodegradability properties that make it a suitable material for encapsulating biological compounds. Initially, pectin was dissolved in a chloroform/methanol solution (1:3 w/w) and then placed in a rotary evaporator (Strik202; Steroglass, Italy) to separate the solvents, resulting in the formation of a thin layer on the wall. Simultaneously, roselle extract was dissolved in a dichloromethane/methanol solution (1:2 w/w), and the resulting mixture was combined with pectin at a ratio of 4:1 (pectin: extract), along with the remaining solvents. The solvent mixture was evaporated under nitrogen vapor. The resulting film was dissolved in 2 mL of phosphate buffer (10 mmol/L, pH 7.4) and homogenized for 15 min at 35°C using a homogenizer (300VT; BioLogics, USA) at a pressure of 200 bar. The obtained suspension was then incubated in the dark at room temperature for 2 h. Subsequently, it was centrifuged (z200A; Hermle, Germany) at 6500 rpm and 4°C. Finally, the nanoencapsulated roselle extract was dried using a freeze dryer (Alpha 2–4 LD Plus; Christ, Germany) (Bahrami Feridoni and Khademi Shurmasti 2020).

### 2.7 Measurement of Particle Size and Zeta Potential

The particle size was measured using a nanoparticle size analyzer (S2‐100‐SZ; Horiba, Japan). The samples were diluted with deionized water at a ratio of 5:1. Additionally, the zeta potential was measured by conducting particle electrophoresis using the same instrument. Prior to analysis, the samples were equilibrated at a temperature of 25°C (Rashidaie Abandansarie, Ariaii, and Charmchian Langerodi 2019).

### 2.8 Encapsulation Efficiency

The encapsulation efficiency (%) of polyphenols was determined using the method described by Jivan, Yarmand, and Madadlou, employing the following formula:

$$100\times \mathrm{EE}\%=\left(C_{e}/C_{t}\right)$$

where C_e_ represents the content of polyphenols released from the capsules (ppm), and C_t_ is the polyphenol content added to the particle formation solution (ppm) (Jivan, Yarmand, and Madadlou 2014).

### 2.9 Bactericidal Assay

The in vitro bactericidal activity of *H. sabdariffa* L. extract was examined against pathogens, including *S. aureus* (ATCC 33591), *S. typhimurium* (ATCC 14028), and *E. coli* (ATCC 1399). These bacterial strains were obtained from the Faculty of Veterinary Medicine, Tehran University, Tehran, Iran, and were prepared from lyophilized stocks.

### 2.10 Determining MIC and MBC

The MIC test was conducted for the selected bacteria using the method recommended by NCCLS. Initially, 0.1 mL of the free and encapsulated extracts of *H. sabdariffa* L. were placed in tubes containing a concentration of 1 × 10^8^ CFU/mL of *S. aureus* (ATCC 33591), *S. typhimurium* (ATCC 14028), and *E. coli* (ATCC 1399) strains. Subsequently, all samples were incubated at 37°C for 24 h. The MIC value was determined as the lowest concentration of *H. sabdariffa* L. extract at which the bacteria exhibited no visible growth. Additionally, the lowest concentration at which no bacteria were detected was identified as the MBC value (Márquez‐Rodríguez et al. 2020).

### 2.11 Sausage Preparation

The general formulation of the produced sausage was in accordance with the usual methods in the industry. A cutter with a temperature of 0°C was used to mix the raw materials listed in Table 1. Initially, ground red meat, salt, and sodium polyphosphate were injected into the cutter (MADO, Germany) with the blades rotating at a speed of 3000 rpm. This mixture was processed for 1.5–2 min. Subsequently, the remaining 1.3% of the formulation, including ice, soy isolate, and ascorbic acid, were added to the cutter, and the processing continued for another 1.5–2 min. After reducing the temperature by two degrees, edible starch, gluten, mixed spices, the remaining 1.3% of ice, and edible liquid oil were added to the cutter. The mixture was sterilized and mixed for 3 min. In separate batches, sodium nitrite and extract, such as free or encapsulated roselle sepals, were added to the dough in different proportions (0:100, 80:20, 60:40, 40:60, 20:80, and 100:0). The remaining ice and water mixture were also added, and the mixture was further processed. Finally, the dough was filled and wrapped in special polyamide covers using a filling machine (SMART, China). The sausages were then heated in a cooking room with steam at 80°C for 60 min, ensuring that the center reached a temperature of 72°C. Subsequently, the sausages were immediately showered with water at 12°C. After cooling, the sausages were stored at room temperature until their temperature reached 4°C. They were then transferred to a refrigerator and stored at this temperature until the experiments were conducted. Throughout the storage period, the sausages were analyzed to determine their microbiological parameters (Khatib et al. 2020). During the cooking and heat treatment of sausages, the bioactive compounds in the sour tea extract may be affected. The phenolic and antioxidant compounds of sour tea extract may decompose due to temperature and heating time. Also, the method of using sour tea extract (free or encapsulated) can affect the stability of these bioactive compounds during the cooking process. Encapsulating the extracts can prevent the degradation of these compounds. Therefore, investigating the effect of the method of using sour tea extract and the thermal parameters of the cooking process on the stability of bioactive compounds in nitrite‐containing sausages will be a suitable topic for future research.

**TABLE 1:** Formulation and ingredients of beef sausage (control treatment).

Row	Components	%
1	Beef sausage	60
2	Water and ice mixture	22
3	Gluten	2
4	Edible starch	5.8
5	Soy isolate	2
6	Salt and spices	2.73
7	Ascorbic acid	0.02
8	Sodium nitrite	0.05
9	Sodium polyphosphate	0.4
10	Liquid oil	5

### 2.12 Microbial Tests Performed on Sausage Samples

To identify microorganisms such as *S. aureus*, *S. typhimurium*, *E. coli*, *coliform*, and *yeast mold* in sausage samples containing nitrite, the free extract of roselle sepals and the enclosed extract of roselle sepals were disinfected with 70% ethanol. The polyamide package was cut by a sterile cutter. Sausage sample (25 g) was added to sterile normal saline solution (225 g) to obtain a 10^−2^ dilution. The sample was homogenized for 2 min under sterile conditions. From this, dilutions of 10^−2^ to 10^−9^ were prepared. All forms were observed on violet red bile agar (VRBA) medium. For Salmonella, Rappaport–Vassiliadis Salmonella enrichment broth (RV broth), tetrathionate broth base (TTB), and brilliant green agar (BGA) were used. And EC broth medium was used to observe *E. coli*. For *S. aureus*, Baird‐Parker Agar (BPA) (incubation at 37°C for 48 h), and for molds and yeasts, Sabouraud dextrose agar (SDA) (incubation at 25°C for 5 days) is used became (Khatib et al. 2020).

### 2.13 Statistical Analysis

The statistical analysis of the results was conducted using SPSS version 22 software. One‐way ANOVA (Analysis of Variance) was used for data analysis and Duncan's multiple range test with a 95% confidence level was used for data comparison. Values with a significance level less than 0.05 were considered statistically significant. Excel software was used for plotting the graphs.

## 3 Results

### 3.1 Measurement of Total Phenol

The amount of total phenolic compounds was determined using the Folin–Ciocalteu reagent, and the calculation was based on milligrams of gallic acid per gram of extract, as described in equation (1). The hydroalcoholic extract contained a total phenol amount of 174.6 mg of gallic acid per gram of the extract.

(1) $$\begin{array}{c} Y=0.0001X+0.4425\\ R^{2}=0.8422 \end{array}$$

### 3.2 Flavonoid Measurement

The amount of flavonoids was determined using the aluminum chloride colorimetric method, and the calculation was based on micrograms of quercetin, as described in equation (2). The hydroalcoholic extract had a flavonoid content of 16.8368 mg/quercetin.

(2) $$\begin{array}{c} Y=0.1348X+2.1152\\ R^{2}=0.8049 \end{array}$$

### 3.3 Assessment of Antioxidant Activity

**FIGURE 1:**
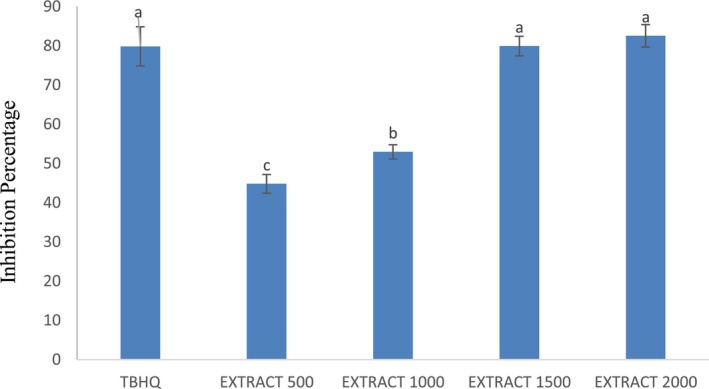
Comparison of different concentrations of roselle extracts on the inhibition rate.

Figure 1 presents the DPPH free radical inhibition rate of roselle extract at different concentrations (500, 1000, 1500, and 2000 ppm) compared to the synthetic antioxidant TBHQ. The highest inhibition rate was observed at the higher concentrations of 1500 and 2000 ppm, as well as with TBHQ. Although there was no significant difference between the concentrations of 1500 and 2000 ppm (*p* ≤ 0.05), these concentrations showed a significant difference when compared to 500 and 1000 ppm of roselle extract (*p* ≥ 0.05) (Figure 1).

### 3.4 Determination of Particle Size, Zeta Potential, and Capsule Efficiency

**FIGURE 2:**
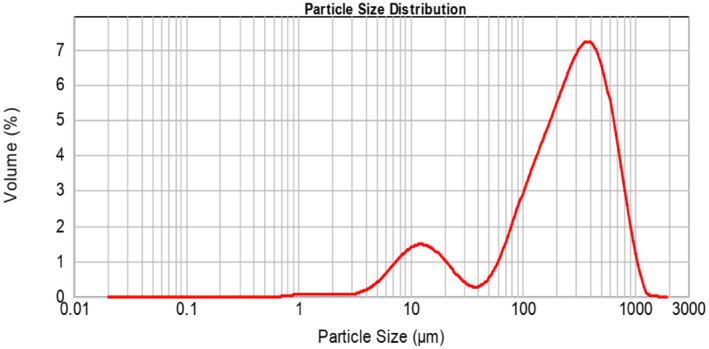
Capsule size.

**FIGURE 3:**
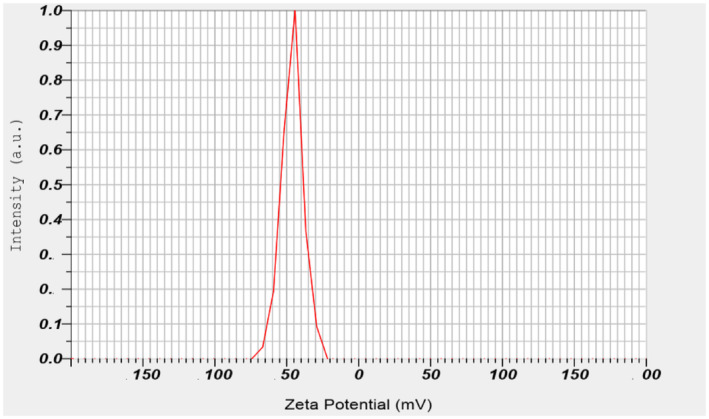
Zeta potential.

Based on the obtained results, the size of the particles encapsulated with pectin ranged from 16.833 to 640.534 μm. The zeta potential of the pectin‐encapsulated extract was measured at −46.1. The efficiency of the produced capsules was determined to be 81.7% (Figures 2 and 3).

### 3.5 MIC and MBC of Free and Encapsulated Extract of Roselle Sepals

The MICs of the free extract of sour tea for *S. aureus*, *S. typhimurium*, and *E. coli* bacteria were determined to be 5, 6, and 5 mg/mL, respectively. In contrast, the MICs of the encapsulated extract of roselle for *S. aureus*, *S. typhimurium*, and *E. coli* bacteria were found to be 3, 4, and 3 mg/mL, respectively (Table 2). Furthermore, the MBCs of the free extract of roselle for *S. aureus*, *S. typhimurium*, and *E. coli* bacteria were observed at 4, 5, and 4 mg/mL, respectively. On the other hand, the MBCs of the encapsulated extract of roselle for *S. aureus*, *S. typhimurium*, and *E. coli* bacteria were determined to be 2, 3, and 3 mg/mL, respectively (Table 3).

**TABLE 2:** Determination of minimum inhibitory concentration (MIC) of free and encapsulated hydroalcoholic extract of roselle sepals.

		MIC (μg/mL)					
Type of extracts	Microorganisms	2	3	4	5	6	Control
Free	*Staphylococcus aureus*	−	−	−	×	−	−
Encapsulated	*Staphylococcus aureus*	−	×	−	−	−	−
Free	*Salmonella typhimurium*	−	−	−	−	×	−
Encapsulated	*Salmonella typhimurium*	−	−	×	−	−	−
Free	*Escherichia coli*	−	−	−	×	−	−
Encapsulated	*Escherichia coli*	−	×	−	−	−	−

*Note:* ×, inhibitory effect; −, being ineffective.

**TABLE 3:** Determination of minimum bactericidal concentration (MBC) of free and encapsulated hydroalcoholic extract of roselle sepals.

Type of extracts	Microorganisms	MBC (μg/mL)					
		2	3	4	5	6	Control
Free	*Staphylococcus aureus*	−	−	×	−	−	−
Encapsulated	*Staphylococcus aureus*	×	−	−	−	−	−
Free	*Salmonella typhimurium*	−	−	−	×	−	−
Encapsulated	*Salmonella typhimurium*	−	×	−	−	−	−
Free	*Escherichia coli*	−	−	×	−	−	−
Encapsulated	*Escherichia coli*	−	×	−	−	−	−

*Note:* ×, lethal effect; −, being ineffective.

### 3.6 Evaluation of the Growth of Microorganisms During the Storage Period in Sausages

In the group of sausages containing nitrite and free extract, no growth of *S. typhimurium*, *E. coli*, and coliform was observed during the storage period. However, *S. aureus* was observed on the 25th and 30th days, while yeast and mold were not observed until the 25th day. However, growth was observed in this group on day 30 (Table 4). In the group of sausages containing the encapsulated extract of roselle sepals, *S. typhimurium* and *E. coli* were not observed at any time. However, *S. aureus* was observed from day 20 onwards, and yeast mold was observed on day 30 (Tables 5 and 6).

**TABLE 4:** Microbial growth in the sausage samples containing nitrite.

Microorganisms	Storage time (days)						
	0	5	10	15	20	25	30
*Staphylococcus aureus*	−	−	−	−	−	×	×
*Salmonella typhimurium*	−	−	−	−	−	−	−
*Escherichia coli*	−	−	−	−	−	−	−
Coliform	−	−	−	−	−	−	−
Yeast mold	−	−	−	−	−	−	×

*Note:* −, no growth of microorganisms; ×, presence of microorganisms.

**TABLE 5:** Microbial growth in the sausage samples containing the free extract of roselle sepals.

Microorganisms	Storage time (days)						
	0	5	10	15	20	25	30
*Staphylococcus aureus*	−	−	−	−	−	×	×
*Salmonella typhimurium*	−	−	−	−	−	−	−
*Escherichia coli*	−	−	−	−	−	−	−
Coliform	−	−	−	−	−	−	−
Yeast mold	−	−	−	−	−	−	×

*Note:* −, no growth of microorganisms; ×, presence of microorganisms.

**TABLE 6:** Microbial growth in the sausage samples containing the encapsulated extract of roselle sepals.

Microorganisms	Storage time (days)						
	0	5	10	15	20	25	30
*Staphylococcus aureus*	−	−	−	−	×	×	×
*Salmonella typhimurium*	−	−	−	−	−	−	−
*Escherichia coli*	−	−	−	−	−	−	−
Coliform	−	−	−	−	−	−	−
Yeast mold	−	−	−	−	−	−	×

*Note:* −, no growth of microorganisms; ×, presence of microorganisms.

## 4 Discussion

### 4.1 Total Phenolic Content and Total Flavonoid Content

Phenolic compounds, including flavonoids, present in fruits and vegetables exhibit strong antioxidant activity, thereby protecting against the dangers of free radicals (Vuolo, Lima, and Junior 2019). In this study, the amount of phenolic compounds in roselle extract was measured as 174.6 mg of gallic acid per gram of extract. Another study reported the amount of phenolic compounds in roselle leaf extract to be 389.98 mg/g, and in sour tea sepal extract to be 474.09 mg/g (Formagio et al. 2015). Bahrami Feridoni and Khademi Shurmasti (2020) also reported the phenolic composition of sour tea extract as 626.57 mg/gallic acid. Additionally, a study reported the total phenolic content of aqueous and alcoholic extracts of roselle to be 77.2 mg/g and 87.7 mg/g, respectively (Al‐Hashimi 2012).

Regarding the measurement of flavonoid content in the hydroalcoholic extract of roselle sepals, the present study reported it as 16.83 mg/quercetin. However, reported the flavonoid content as 12.30 ± 0.09 mg/g in their research (Builders et al. 2013). Formagio et al. (2015) observed the highest amount of flavonoids in sour tea leaf extract as 104.52 mg/g, and in roselle sepal extract as 148.35 mg/g.

### 4.2 Investigating DPPH Free Radical Activity

The DPPH assay is a reliable, accurate, and cost‐effective method for evaluating the antioxidant properties of essential oils and plant extracts in the laboratory. This test measures the ability of compounds to donate a hydrogen or electron to the stable DPPH radical, causing it to be reduced and change color (Echegaray et al. 2021). The results of this study showed that increasing the concentration of compounds like TBHQ and sour tea extract significantly enhanced their DPPH radical inhibition rate. These natural antioxidants demonstrated a high potential to prevent oxidation and spoilage in sausages, and their DPPH radical scavenging ability was better than that of synthetic antioxidants. Therefore, the findings of this DPPH assay can inform the development of natural preservative systems to improve the shelf life of sausages. Overall, the DPPH test provides a useful and reproducible method for assessing the antioxidant capacities of various plant‐derived compounds in a laboratory setting. Similarly, Fereydoni's study found that the DPPH free radical inhibition was influenced by different concentrations of the extract, increasing with higher extract concentrations. The highest DPPH radical inhibition activity was observed at a concentration of 1000 ppm (87.86%) (Bahrami Feridoni and Khademi Shurmasti 2020). Furthermore, research results indicated that the DPPH values for sour tea infusion extract and ethanol extract were measured as 0.88 mg/mL and 3.78 mg/mL, respectively (Sartini et al. 2020). Another study showed that the antioxidant activity of the alcoholic extract of roselle was equivalent to the artificial antioxidant BHT (75.67%), with the alcoholic extract demonstrating the highest regenerative ability (Al‐Hashimi 2012). Additionally, Hamrita et al. (2022) demonstrated in their study that both methanolic and aqueous extracts of the calyx of roselle exhibited the capability to eliminate the DPPH free radical to varying degrees, with an efficacy of up to 86%. The antioxidant property of sour tea extract was also reported in the studies of (Formagio et al. 2015 and Jafarian et al. 2014).

### 4.3 Determination of Optimal Capsule Particle Size and Determination of Zeta Potential

In the encapsulation process of roselle extract (*H. sabdariffa* L.) using pectin as an encapsulating agent, the observed particle size ranged from 16,833 to 640,534 μm. The zeta potential of −46.1 mV indicated a stable colloidal system with a wide distribution of particle sizes. The wide range of particle sizes can be attributed to the variable encapsulation abilities of pectin, which can be customized for specific food applications. The very negative zeta potential indicates that the particles are well dispersed and resistant to agglomeration due to electrostatic repulsion. This property is beneficial for the uniformity and stability of the encapsulated extract in various food matrices, such as sausages. The stability is necessary to ensure the longevity and effectiveness of the antioxidant properties of the encapsulated extract, ultimately increasing the nutritional value and shelf life of the final food product. In a study, it was shown that roselle extract was nanoencapsulated using maltodextrin–milk protein concentrate, resulting in a particle size of 139.03 ± 2.76 nm (Bahrami Feridoni and Khademi Shurmasti 2020).

### 4.4 Capsule Efficiency

According to the results of the present study, the capsules produced using pectin exhibited an efficiency of 81.7%. This finding indicates that the use of pectin as an encapsulation agent can effectively protect roselle extract and preserve its active ingredients. Furthermore, in Faridooni's study, it was found that the efficiency of nanoencapsulation of roselle extract using maltodextrin–milk protein concentrate complex was measured as 67.37% ± 1.37%, with the nanoencapsulated extract demonstrating high efficiency (Bahrami Feridoni and Khademi Shurmasti 2020).

### 4.5 Microbial Evaluation of Free and Encapsulated Extracts

The encapsulated roselle extract demonstrated superior antibacterial properties compared to the free extract, as evidenced by its lower MIC and MBC values. The MIC results showed the encapsulated extract exhibited stronger inhibitory effects on the growth of the tested pathogenic bacteria, including *S. aureus*, *S. typhimurium*, and *E. coli*. Specifically, the encapsulated extract had lower MIC values than the free extract against all three bacteria, indicating it could inhibit their growth at lower concentrations. Furthermore, the MBC results further supported the enhanced efficacy of the encapsulated extract, as it exhibited lower MBC values compared to the free extract for *S. aureus*, *S. typhimurium*, and *E. coli*. The lower MBC values not only demonstrate the encapsulated extract's ability to inhibit bacterial growth but also its improved capacity to kill the bacteria at lower concentrations. The improved antibacterial activity of the encapsulated roselle extract can be attributed to the protective barrier provided by the encapsulation process, which may prevent degradation of the active compounds and ensure their sustained delivery to the site of action. These findings suggest the encapsulated roselle extract has great potential as a natural antimicrobial agent for enhancing the microbiological safety and extending the shelf life of sausages and other food products. In the current study, the antibacterial effect of free and encapsulated roselle extract (*H. sabdariffa* L.) was investigated against pathogenic bacteria, including *S. aureus*, *S. typhimurium*, and *E. coli*. The MIC and MBC were used to evaluate the potential of roselle as a natural antimicrobial agent. The MIC results demonstrated that the encapsulated extract of roselle exhibited a better inhibitory effect on the growth of the tested bacteria compared to the free extract. Specifically, the encapsulated extract had a lower MIC than the free extract against *S. aureus*, *S. typhimurium*, and *E. coli*. This suggests that the encapsulation process may increase the bioavailability and stability of the bioactive compounds of roselle, resulting in stronger antibacterial activity. Furthermore, the MBC results further supported the increased efficacy of the encapsulated extract against *S. aureus*, *S. typhimurium*, and *E. coli* compared to the free extract. The lower MBC values of the encapsulated extract not only indicate its ability to inhibit bacterial growth but also to kill bacteria at lower concentrations. The observed increase in antibacterial activity of the encapsulated roselle extract can be attributed to the protective barrier provided by the capsule, which may prevent degradation of the active compounds and ensure their sustained delivery to the site of action. Also, the lower MIC and MBC values of the encapsulated roselle extract can help protein manufacturers, especially sausages, use lower concentrations of the encapsulated extract to achieve the same level of antimicrobial protection, which leads to increased food safety, increased shelf life, and reduced cost will be on the other hand. Our findings are consistent with other studies that have reported the benefits of encapsulating plant extracts with antimicrobial properties. For example, Zhang et al. (2022) demonstrated in their research that encapsulated plant extracts exhibit higher stability and improved antimicrobial activity against various microorganisms. Another study examined the antimicrobial activity of calyx extract from five cultivars of *H. sabdariffa* against *S. typhimurium* and *Salmonella chleraesuis*. The extracts produced with water, ethanol, and methanol showed antimicrobial activity against both serotypes of Salmonella, suggesting the antimicrobial effect of the extract. The variety of *H. sabdariffa* calices was found to impact the antimicrobial activity (Morales‐Cabrera et al. 2013). Furthermore, research has shown the potential use of roselle extract as a source of plant compounds with antimicrobial and antioxidant activities (Hamrita et al. 2022). In Javadian et al.'s (2015) study, the ethanolic extract of roselle exhibited the highest inhibitory effect (MIC) against *K. pneumoniae* samples at concentrations of 11 and 5 mg/mL. Additionally, the research results indicated that roselle extract significantly reduced the number of *S. aureus* in the production of smoked beef, although no reduction was observed in samples containing *E. coli*, coliform extracts, and *Salmonella* (Malelak, Sipahelut, and Jelantik 2017).

### 4.6 Evaluation of Microbial Characteristics of Sausage Samples

The present study also assessed the effectiveness of nitrite, free roselle sepal extract, and encapsulated roselle sepal extract in inhibiting microbial growth in beef sausage during storage. The results indicated that sausages treated with nitrite and free roselle sepal extract exhibited no growth of *S. typhimurium*, *E. coli*, or coliform microorganisms during the storage period, indicating the strong inhibitory effects of these compounds.

The effectiveness of nitrites in preventing the growth of various pathogenic bacteria in meat products and their well‐established antimicrobial effect have been extensively demonstrated. On the other hand, roselle sepal extract may contain bioactive compounds that contribute to microbial inhibition. In our study, *S. aureus* was detected on days 25 and 30, along with delayed growth of yeasts and molds. However, this growth only occurred on day 30. These results indicate that the additives have a significant inhibitory effect on certain bacteria but are less effective against other microorganisms such as *S. aureus*. Additionally, the presence of yeasts and molds may be limited to a specific time frame.

Previous studies have shown that the delayed onset of microbial growth aligns with the effect of antimicrobial agents in food products. This suggests that antimicrobial agents can extend the lag phase of microbial populations in food products but may not completely inhibit their growth indefinitely (Papadochristopoulos et al. 2021). In contrast, our study revealed the detection of *S. aureus* in sausages containing encapsulated roselle sepal extract on the 20th day, indicating a reduced antimicrobial effect compared to the free extract. The observed differences may be attributed to factors such as the encapsulation technique employed and the interaction between the encapsulated compounds and the meat matrix, which can influence the biological activity of the antimicrobial agents (Bahrami Feridoni and Khademi Shurmasti 2020; Zhang et al. 2022). The growth of yeasts and molds was observed until day 30 in both groups, indicating the need for a comprehensive strategy for microbial protection and control. Although additives have demonstrated promising results against bacteria, their limited effect on yeasts and molds necessitates the use of additional or supplementary preservation methods to ensure the microbial integrity and quality of sausages throughout their shelf life.

The antimicrobial effect of roselle sepal extract on beef sausage is likely to involve several factors. Roselle sepal extract contains bioactive compounds such as phenolic compounds and flavonoids, which are known for their antioxidant and antimicrobial properties (Borrás‐Linares et al. 2015; Jung, Kim, and Joo 2013). These compounds have been shown to inhibit the growth of certain pathogenic bacteria (Hamrita et al. 2022; Javadian et al. 2015; Morales‐Cabrera et al. 2013). Additionally, the encapsulation process increases the bioavailability and stability of these bioactive compounds, resulting in stronger antibacterial activity (García, Forbe, and Gonzalez 2010; Neethirajan and Jayas 2011; Zhang et al. 2022). The encapsulated roselle sepal extract forms a protective barrier around the bioactive compounds, preventing their degradation. This protective barrier also controls the release rate of bioactive compounds, prolonging their antimicrobial activity (Bahrami Feridoni and Khademi Shurmasti 2020). Moreover, the encapsulated extract exhibits a wide distribution of particle sizes and a negative zeta potential, indicating a stable colloidal system. Therefore, in the present study, both the free and encapsulated extracts of roselle are likely to enhance the nutritional value and extend the shelf life of the final food product by virtue of their antimicrobial properties, conducting more research on optimizing the encapsulation methods of roselle petal extract can lead to improving its antimicrobial efficiency and stability in meat products. Also, investigating the use of other natural extracts or their combinations with complementary properties will provide new ways to develop effective natural preservatives for meat. Screening a wider range of plant sources and evaluating their characteristics can also help to expand sustainable and healthy options to maintain meat quality.

## 5 Conclusion

In summary, the current research evaluating the microbial characteristics of beef sausage enriched with roselle sepal extract (*H. sabdariffa* L.) demonstrates promising results. The encapsulated roselle sepal extract exhibits enhanced antimicrobial activity against *S. aureus*, *S. typhimurium*, and *E. coli* compared to the free extract. The encapsulation process leads to stronger inhibitory effects on bacterial growth. Additionally, the encapsulated extract demonstrates stable colloidal properties, characterized by a wide particle size distribution and negative zeta potential. This stability ensures uniformity and consistency within the sausage matrix, preserving the antioxidant properties of the encapsulated extract and extending the product's shelf life. Although this research had limitations in investigating the effect of roselle extract on certain characteristics of beef sausage, its findings highlight the potential of roselle sepal extract as a natural antimicrobial agent in beef sausage production. The encapsulation of the extract enhances its efficiency and renders it a valuable ingredient in food formulations aimed at improving food safety and quality. It also allows for significant improvement in the microbial control of beef sausages. Therefore, the use of this natural extract can act as an effective strategy for sausage producers to increase the shelf life and improve the quality of their products. However, further research is necessary to optimize the encapsulation parameters and explore the sensory characteristics and consumer acceptance of sausages enriched with roselle.

## Author Contributions

**Sharif Ghorbani:** investigation (equal), methodology (equal), writing – original draft (equal). **Sara Jafarian:** project administration (equal), supervision (equal). **Mahdi Sharifi Soltani:** conceptualization (equal), writing – review and editing (equal). **Leila Roozbeh Nasiraie:** conceptualization (equal), writing – review and editing (equal).

## Conflicts of Interest

The authors declare no conflicts of interest.

## Data Availability

The data that support the findings of this study are available on request from the corresponding author.
